# Soft Tissue Sarcomas with Chromosomal Alterations in the 12q13-15 Region: Differential Diagnosis and Therapeutic Implications

**DOI:** 10.3390/cancers16020432

**Published:** 2024-01-19

**Authors:** Javier Lavernia, Reyes Claramunt, Ignacio Romero, José Antonio López-Guerrero, Antonio Llombart-Bosch, Isidro Machado

**Affiliations:** 1 Oncology Unit, Instituto Valenciano de Oncología, 46009 Valencia, Spain; jlavernia@fivo.org; 2 Laboratory of Molecular Biology, Instituto Valenciano de Oncología, 46009 Valencia, Spain; rclaramunt@fivo.org (R.C.); jalopez@fivo.org (J.A.L.-G.); 3 Pathology Department, University of Valencia, 46010 Valencia, Spain; antonio.llombart@uv.es; 4 Pathology Department, Instituto Valenciano de Oncología, 46010 Valencia, Spain; 5 CIBERONC Cancer, 28029 Madrid, Spain; 6 Patologika Laboratory, Hospital Quiron-Salud, 46010 Valencia, Spain

**Keywords:** *MDM2*, *CDK4*, *GLI1*, liposarcomas, *GLI1*-altered neoplasm, FISH, MDM2/CDK4 inhibitors

## Abstract

**Simple Summary:**

The chromosomal region 12q13-15 is rich in oncogenes (*MDM2*, *CDK4*, *STAT6*, *DDIT3*, and *GLI1*). Amplification of *MDM2* and *CDK4* genes can be detected in various mesenchymal and nonmesenchymal neoplasms. Therefore, gene amplification alone is not entirely specific for making a definitive diagnosis and requires the integration of clinical, radiological, morphological, and immunohistochemical findings. Despite the diagnostic implications that the overlap of genetic alterations in neoplasms with changes in genes within the 12q13-15 region could create, the discovery of coamplifications of *MDM2* with *CDK4* and *GLI1* offers new therapeutic targets in neoplasms with *MDM2/CDK4* amplification. In this review, we delve into the diagnosis and therapeutic implications of neoplasms with genetic alterations involving the chromosomal region 12q13-15, mainly *MDM2*, *CDK4*, and *GLI1*.

**Abstract:**

The chromosomal region 12q13-15 is rich in oncogenes and contains several genes involved in the pathogenesis of various mesenchymal neoplasms. Notable genes in this region include *MDM2*, *CDK4*, *STAT6*, *DDIT3*, and *GLI1*. Amplification of *MDM2* and *CDK4* genes can be detected in various mesenchymal and nonmesenchymal neoplasms. Therefore, gene amplification alone is not entirely specific for making a definitive diagnosis and requires the integration of clinical, radiological, morphological, and immunohistochemical findings. Neoplasms with *GLI1* alterations may exhibit either *GLI1* rearrangements or amplifications of this gene. Despite the diagnostic implications that the overlap of genetic alterations in neoplasms with changes in genes within the 12q13-15 region could create, the discovery of coamplifications of *MDM2* with *CDK4* and *GLI1* offers new therapeutic targets in neoplasms with *MDM2/CDK4* amplification. Lastly, it is worth noting that *MDM2* or *CDK4* amplification is not exclusive to mesenchymal neoplasms; this genetic alteration has also been observed in other epithelial neoplasms or melanomas. This suggests the potential use of *MDM2* or *CDK4* inhibitors in neoplasms where alterations in these genes do not aid the pathological diagnosis but may help identify potential therapeutic targets. In this review, we delve into the diagnosis and therapeutic implications of tumors with genetic alterations involving the chromosomal region 12q13-15, mainly *MDM2*, *CDK4*, and *GLI1*.

## 1. Introduction

Soft tissue sarcomas are a group of predominantly aggressive malignant tumors. The incidence is 5 cases per 100,000 inhabitants per year in Europe, representing less than 1% of all malignant tumors [1]. They share the common characteristic of originating from mesenchymal tissues.

Soft tissue sarcomas affect individuals throughout the entire age range, and important differences exist in the presentation of these sarcomas across varying age groups [2]. Notably, rhabdomyosarcoma or Ewing sarcoma is more common in children, while most nonrhabdomyosarcoma soft tissue sarcoma subtypes are more prevalent in adults [2]. Despite their rarity, sarcomas carry a large disease burden in the pediatric and adult populations and are a significant cause of cancer deaths during the first 20 years of life [2]. There are numerous genetic syndromes that increase the risk of developing sarcomas, including neurofibromatosis type 1, Maffucci’s syndrome, Li–Fraumeni syndrome, and McCune–Albright syndrome [2,3,4,5]. In addition, exposure to radiation is an established risk factor for sarcomas, and immune suppression has also been implicated in the development of sarcomas [2,3,4,5]. Multiple environmental risk factors for sarcoma development exist, but drawing definitive conclusions regarding the association between risk factors and sarcomas have proven to be challenging [3,4,5]. Soft tissue sarcomas may arise in extremities, abdomen/retroperitoneum, trunk, head, and neck area [2,3,4,5]. The majority of patients with soft tissue sarcomas present with a painless mass, although pain is noted at presentation in up to a third of cases [2,3,4,5]. Delay in the diagnosis of sarcomas is common, with the most common incorrect diagnosis for extremity and trunk lesions being hematoma, cystic, or benign adipocytic tumor [3,4,5]. Late diagnosis of retroperitoneal sarcomas is common because tumors in this area can grow to massive size before causing any symptoms. In large tumors, patients may complain of abdominal distention or discomfort [3,4,5]. From an epidemiological point of view, little information is readily available on patterns of incidence and survival in specific geographic areas for sarcomas [2,3,4,5].

Tumors classified under the term ‘soft tissue sarcomas’ encompass a highly heterogeneous group of different pathologies, leading to significant diagnostic and therapeutic complexity [6,7,8,9,10,11,12,13]. Diagnosing soft tissue sarcomas necessitates specific techniques such as immunohistochemistry (IHC), in situ hybridization, and molecular biology ancillary tests, often resulting in distinct therapeutic approaches based on histotype variations [6,7,8,9,10,11,12,13].

According to the WHO classification, over 100 different histological types of soft tissue sarcomas are categorized under the term ‘soft tissue sarcomas’ [6,7]. They exhibit substantial clinical and pathological heterogeneity, presenting a diagnostic challenge for pathologists who often rely on complementary immunohistochemical and molecular biology studies to identify molecular alterations [6,7,8,9,10,11,12,13,14,15,16,17]. These alterations can be highly valuable for implementing targeted therapies aimed at improving treatment effectiveness and enhancing patient survival and quality of life [17].

The low incidence of these tumors may lead to discrepancies between pathological diagnoses and IHC or molecular results [14,15,16,17]. Consequently, in recent years, it has been recommended that sarcoma diagnoses be confirmed by pathologists with expertise in this field [14,15]. These pathologists have benefited from significant advancements in molecular biology, enabling more precise diagnoses of various histological types of sarcomas, particularly those necessitating molecular techniques for diagnostic confirmation. Similarly, it is advisable that the diagnosis and treatment of sarcomas be conducted in specialized centers with multidisciplinary teams dedicated to this pathology [16].

Frequently, we encounter situations where sarcomas exhibit significant similarities not only among different histological types of sarcomas but also with other neoplasms like melanomas, carcinomas, or mesotheliomas [6,7,8]. Therefore, accurate diagnosis and potential therapeutic implications are crucial. For instance, distinguishing between an epithelioid melanoma and a clear cell sarcoma (both expressing S100 and melanocytic markers) or a spindle cell/desmoplastic melanoma and a malignant peripheral nerve sheath tumor (both expressing S100 and SOX10) is challenging [18,19,20,21,22]. In such cases, while molecular biology can provide essential data, such as the presence of *BRAF*, *KIT*, or *TERT* mutations commonly found in melanoma and the presence of *EWSR1::ATF1*/*CREB1* rearrangement favoring clear cell sarcoma, clinical-pathological correlation remains indispensable [18,19,20,21,22].

In the context of a prior diagnosis of melanoma, the possibility of dedifferentiated melanoma should always be excluded, as the morphology may be indistinguishable from that of a sarcoma. Conversely, when faced with a diagnostic challenge between spindle cell melanoma and neural sarcoma, clinical data such as location in sun-exposed areas of the skin can indicate a higher likelihood of spindle cell melanoma [18,19,20,21,22].

It is crucial to emphasize that any molecular result must be closely correlated with clinical-pathological findings. Many genes can be altered (mutations, rearrangements, etc.) and are not specific [6,7,8,18,19,20,21,22]. For instance, the *EWSR1* gene or the *MDM2* gene can be rearranged or amplified in various types of sarcomas, carcinomas, or even mesotheliomas [6,7,8]. Therefore, an isolated molecular finding should not be interpreted as defining a specific diagnosis.

The present review describes the diagnosis and therapeutic implications of tumors with genetic alterations involving the chromosomal region 12q13-15, mainly *MDM2*, *CDK4,* and *GLI1.*

## 2. What Do We Know about the MDM2 Gene?

**Figure 1:**
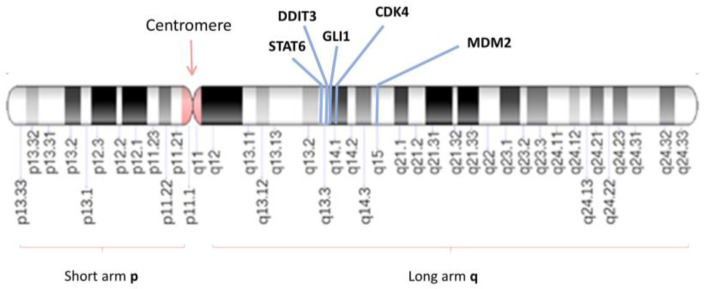
The genomic positions and relationship of the genes located on the 12q13-15 locus. G-banding patterns of human chromosome 12 with a resolution of 850 bands. The band length in this diagram is based on the ISCN (2013) ideograms. The chromosomal position of the *STAT6* (12q13.3), *DDIT3* (12q13.3), *GLI1* (12q13.3), *CDK4* (12q14q.1) and *MDM2* (12q15) genes are indicated by blue lines. ISCN: The International System for Human Cytogenomic Nomenclature; STAT6: Signal transducer and activator of transcription 6; DDIT3: DNA damage-inducible transcript 3; GLI1: glioma-associated oncogene 1; CDK4: *Cyclin Dependent Kinase 4*; *MDM2*: *Mouse double minute 2 homolog*.

**Figure 2:**
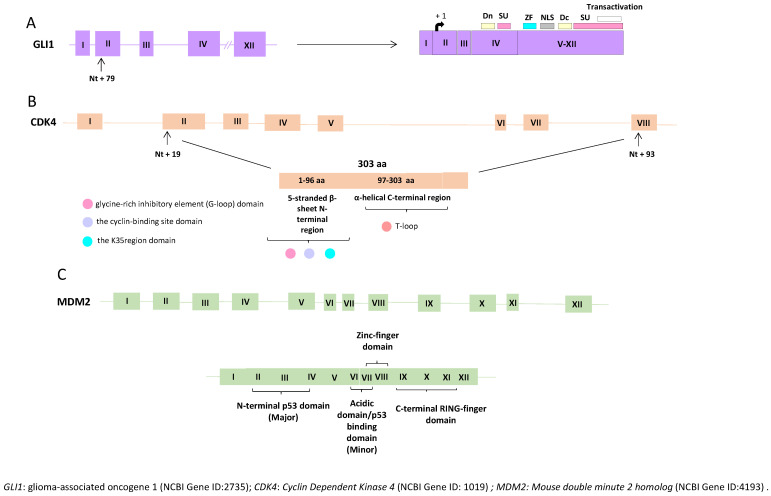
Structures and the encoded full-length of the human *GLI1*, *CDK4,* and *MDM2* genes. (**A**) The full-length *GLI1* gene comprises 12 exons, including the 5′-untranslated exon 1. The GLI isoform 1 coding region starts at nt +79 in exon 2 (arrow). The known functional domains of full-length *GLI1* include the degron degradation signals (Dn and Dc; yellow boxes), SUFU-binding domains (SU; pink boxes), zinc finger domains (ZF; blue box), the nuclear localization signal (NLS; grey box), and the transactivation domain (white box). (**B**) The full-length *CDK4* gene comprises eight exons. The CDK4 open-reading frame (ORF) involves a start codon that is located 19 nt from the 5′end of exon 2 and a stop codon that resides 93 nt from the 5′end of exon 8 (arrow). The first 1–96 aa residues of the CDK4 protein form the 5-stranded β-sheet N-terminal region, while the remaining residues (97–303 aa) compose the mainly α-helical C-terminal region (indicated by orange boxes). The N-terminal contains three significant domains, the cyclin-binding site domain, the glycine-rich inhibitory element (G-loop), and the K35 region. The C-terminal (residues 97–303) contains regions and domains crucial for the activation of the protein. (**C**) The full-length *MDM2* gene comprises 12 exons, and the relevant protein domains are indicated. Exons are depicted as boxes, and introns as lines.

The *MDM2* gene (short for ‘murine double minute 2′) is an oncogene located on the long arm of chromosome 12 at cytoband q15 (12q15), responsible for encoding the MDM2 protein [23,24,25,26,27] (Figure 1 and Figure 2). Discovered in 1987, the *MDM2* gene originated from a transformed mouse cell line (3T3-DM) [23].

The protein product of the *MDM2* gene is the MDM2 protein, which serves as a significant negative regulator of the tumor suppressor p53. MDM2 is known to interact with p53, repressing its transcriptional activity by binding to and blocking the N-terminal transactivation domain of p53 (Figure 2). Additionally, MDM2 functions as an E3 ubiquitin ligase, recognizing the N-terminal transactivation domain (TAD) of the p53 protein. In this manner, MDM2 targets p53 for ubiquitination and transports it to the cytoplasm for degradation in the proteasome [24,25,26,27].

Structurally and functionally, the *MDM2* gene encodes a 491-amino acid protein with a molecular weight of 56 kDa (Figure 2). This protein contains various conserved structural domains, including the p53 interaction domain at the N-terminus, a central acidic domain, and a zinc finger domain. MDM2 also features a RING domain at the C-terminus, conferring E3 ubiquitin ligase activity on MDM2 [27,28,29,30].

**Figure 3:**
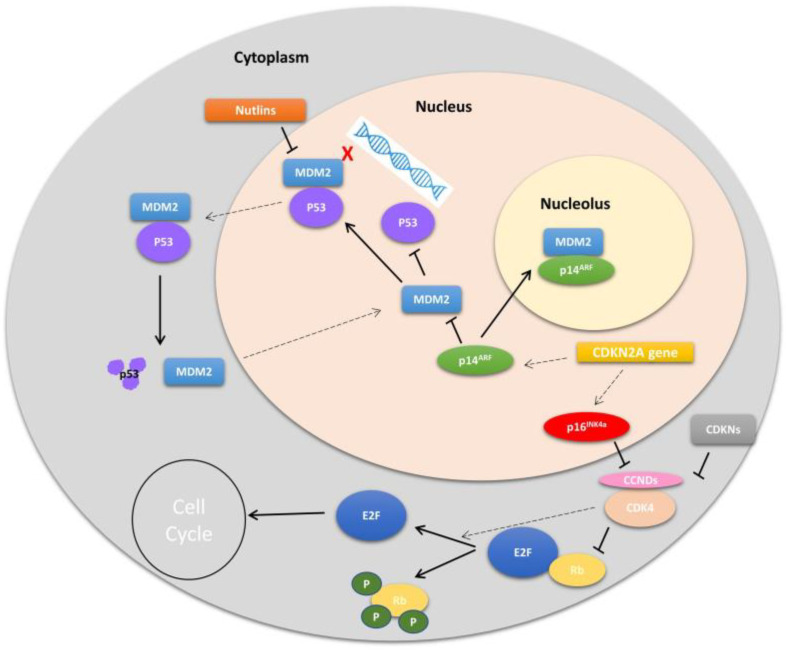
Overview of MDM2 and CDK4 pathway regulation. One of the main roles of *MDM2* is through its direct binding and inhibition of TP53-mediated activation of cell cycle arrest and apoptosis. Nutlins inhibit MDM2-TP53. Active CDK4 in complex with D-type cyclins (CCNDs) hyper-phosphorylates Rb causing release of E2F to promote cell proliferation. *MDM2*: *Mouse double minute 2 homolog*; *CDK4*: *Cyclin Dependent Kinase 4*; *TP53*: *tumor protein 53*; *CCNDs*: *D-type cyclins*; *Rb*: *Retinoblastoma protein*; *E2F*: *family of transcription factors*; *CDKN2A gen*: *cyclin-dependent kinase inhibitor 2A p16INKAa*: *cyclin-dependent kinase inhibitor 2A protein*; *p14ARF*: *ARF tumor suppressor (alternate reading frame protein product of the CDKN2A locus)*.

Several known mechanisms regulate MDM2. One of these mechanisms involves the phosphorylation of the MDM2 protein. Phosphorylation of MDM2 occurs when DNA damage is detected, leading to changes in the function and stability of p53. Additionally, phosphorylation of specific residues in the central acidic domain of MDM2 can enhance its ability to target p53 for degradation (Figure 3). Another mechanism for negatively regulating the p53-MDM2 interaction is the induction of the p14arf protein, a product of an alternative reading frame of the *CDKN2A* locus (p16INK4a/ARF). The p14arf protein directly interacts with MDM2, resulting in the activation of the transcriptional response of p53. ARF sequesters MDM2 in the nucleolus, inhibiting nuclear export and activating p53, as proper p53 degradation depends on its transport to the nucleus [30,31].

The levels and stability of MDM2 are also regulated through ubiquitination. MDM2 undergoes auto-ubiquitination, enabling its degradation by the proteasome. Additionally, MDM2 interacts with a specific ubiquitin protease, USP7, which can reverse MDM2 ubiquitination and prevent its degradation by the proteasome. It is worth noting that USP7 also protects p53 from degradation, given that p53 is the primary target of MDM2 [32]. As a result, MDM2 and USP7 form a complex circuit to finely regulate p53 stability and activity. Maintaining stable levels of p53 is crucial for the correct p53 function [32].

The overall frequency of *MDM2* gene amplification in human cancer varies between 3.5% and 4.4% [33,34]. *MDM2* amplification has been reported in some gliomas, carcinomas, and hematological neoplasms, but this characteristic is much more frequently observed in sarcomas.

Early cytogenetic studies that characterized chromosomal abnormalities in soft tissue sarcomas identified recurrent alterations associated with the 12q13-14 locus [35]. After the characterization and localization of the *MDM2* gene on chromosome 12 [36], a study was conducted with different histological subtypes of sarcomas, revealing the presence of *MDM2* amplification in osteosarcoma, liposarcoma, lipoma, leiomyosarcoma, rhabdomyosarcoma, malignant schwannoma, fibrosarcoma, hemangiopericytoma, and malignant fibrous histiocytoma [33,34,35,36,37,38,39,40,41,42]. Furthermore, it was demonstrated that *MDM2* amplification was associated with the overexpression of both RNA and proteins [37,41,43]. This amplification occurred mutually exclusively of *p53* mutation [39,41,44].

In summary, these findings supported the developing hypothesis that high MDM2 expression through gene amplification represented an alternative mechanism to *p53* mutation for inactivating the p53 signaling pathway and promoting tumor progression in sarcomas.

In soft tissue sarcomas, the amplification of *MDM2* primarily occurs through the mechanism known as double minute chromosomes (Dmins) [45]. Dmins are small chromatin bodies, typically acentric, serving as an amplification mechanism for various oncogenes, including *MDM2* [46,47,48,49]. Sarcomas that exhibit the highest percentages of *MDM2* amplification include low-grade/periosteal osteosarcoma, atypical liposarcoma/lipomatous tumor, dedifferentiated liposarcoma, and intimal sarcoma [7,8,9,50]. Mixofibrosarcomas, malignant peripheral nerve sheath tumors, and undifferentiated sarcomas can also occasionally display *MDM2* amplification. Recently, a rare subtype of endometrial sarcoma characterized by a *BCOR* rearrangement was reported to have *MDM2* amplifications [51]. *MDM2* amplification is present in 95% of well-differentiated and dedifferentiated liposarcomas, while benign lipomatous lesions do not show any amplification; thus, evaluating *MDM2* status is crucial in the diagnosis of liposarcoma [52,53].

*MDM2* gene amplifications can be detected using various techniques, including fluorescence in situ hybridization (FISH), qualitative PCR, comparative genomic hybridization (CGH), immunohistochemistry (IHC), and chromogenic in situ hybridization (CISH/DISH) [50,51,52,53,54,55]. Generally, IHC and FISH techniques are widely employed in most centers. However, it has been noted in various studies that there may not be a strong correlation between IHC (protein overexpression of MDM2) and FISH (*MDM2* gene amplification) in certain histological subtypes of liposarcomas, especially in cases with poor differentiation or MDM2 overexpression unrelated to gene amplification [50,51,52,53,54,55]. Therefore, considering the clinical implications of misdiagnosing these lesions, molecular analysis of *MDM2* is necessary and is often performed using fluorescence in situ hybridization (FISH), which is considered the gold-standard technique [50,51,52,53,54,55].

**Figure 4:**
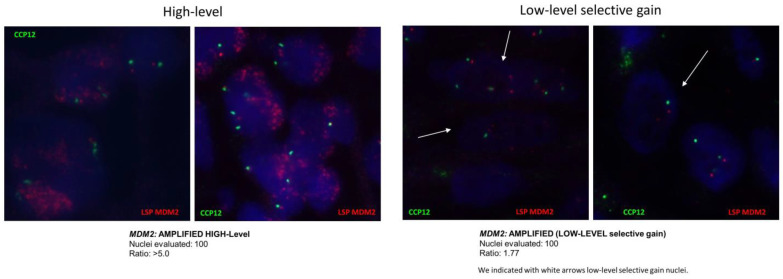
*MDM2* amplification pattern by FISH (high level and *MDM2* gain). Magnification: 63×.

For the study of *MDM2* gene amplifications, the FISH technique is conducted using a combination of two FISH probes. One probe marks the centromere of chromosome 12, serving as a control for ploidy (a control probe to determine the number of copies of chromosome 12 per cell), while the other probe marks the region of the gene of interest (*MDM2*). This results in a ratio between the number of specific *MDM2* signals and the centromeric signals. Different levels of amplification have been described based on this ratio (Figure 4):

1. High-level amplification (HIGH-LEVEL): A ratio equal to or greater than 5.0 in at least 10% of the analyzed nuclei.
2. Low-level amplification (LOW-LEVEL): A ratio equal to or greater than 2.0 in at least 20% of the analyzed nuclei.
3. Low-level selective gain: If the ratio is equal to or greater than 1.5 in at least 20% of the analyzed nuclei, it is considered low-level selective gain.
4. Not amplified: If the ratio is less than 1.5 in over 80% of the analyzed nuclei [52,53,54,55,56,57,58,59].

## 3. The *CDK4* Gene Is a Close Neighbor of the *MDM2* Gene

The *CDK4* gene is located on chromosome 12 at cytoband q14, distal to the *GLI1* and *CHOP/DDIT3* genes situated on 12q13.3-q14.1 (Figure 1), and proximal to *MDM2*, located on 12q14.3-q15 [60,61,62]. Structurally, the *CDK4* gene is composed of eight exons and has a size of 5 Kb, encoding the CDK4 protein consisting of 303 amino acids (Figure 2) [60,61,62].

The *CDK4* gene encodes a protein-serine kinase called cyclin-dependent kinase 4 (CDK4), which plays a role in the cell cycle (Figure 3). Cell division in humans is primarily regulated at the transitions between the cell cycle phases G1–S or G2–M. Specifically, CDK4 activity is restricted to the G1–S phase transition and is controlled as follows: (1) positively by its association with regulatory cyclin D subunits (D1, D2, and D3), and (2) negatively by tumor suppressors such as p16INK4A encoded by CDKN2A, which prevents the interaction of CDK4 with cyclin Ds [63]. Cyclin D and CDK4 complexes phosphorylate proteins involved in the control of cell proliferation during the G1 phase, such as the Rb (retinoblastoma) protein. Due to the critical roles of both p16INK4A and Rb in the regulation of cell proliferation, inactivating mutations and deletions in the genes encoding these regulators are common in many types of tumors [64,65,66]. Additionally, CDK4 also phosphorylates other proteins such as FOXM1, NFAT4, and SMAD3 [67,68,69].

Mutations in the *CDK4* gene, as well as its associated proteins (cyclin Ds, p16INK4A, and Rb), are associated with carcinogenesis in various organs. In addition, *Cyclin D* gene overexpression has been described in many cancers, including those affecting the breast, esophagus, liver, and a subgroup of lymphomas. Furthermore, *CDK4* gene amplification is also found in melanomas, sarcomas, and glioblastomas [53,70]. Therefore, despite *CDK4* amplification and/or Cyclin D overexpression having therapeutic implications, both alterations are entirely nonspecific to any particular diagnosis, and a thorough clinical, histological, and phenotypic correlation should be carried out.

Similar to the detection of *MDM2* gene amplifications, the most widely used techniques for detecting *CDK4* gene amplifications are IHC (immunohistochemistry) and FISH (fluorescence in situ hybridization). For the study of *CDK4* gene amplifications, the FISH technique is performed using a combination of two FISH probes [53,70]. One probe marks the centromere of chromosome 12, serving as a control for ploidy (a control probe to determine the number of chromosome 12 copies per cell), and the other probe marks the region of interest (*CDK4*). Amplification of *CDK4* is considered in cases where the ratio is greater than 2.0, considering the following signal pattern per cell: 2–4 CCP12 signals/>6 CDK4 signals [53,70].

## 4. The *GLI1* Gene Is Also in the Vicinity of *MDM2* and *CDK4*

The *GLI1* gene encodes the oncogenic protein associated with glioma, known as the Zinc Finger Protein GLI1 (68). The *GLI1* gene is located on chromosome 12 at cytoband q13, specifically q13.2-q13.3 (Figure 1 and Figure 2) [71,72,73].

**Figure 5:**
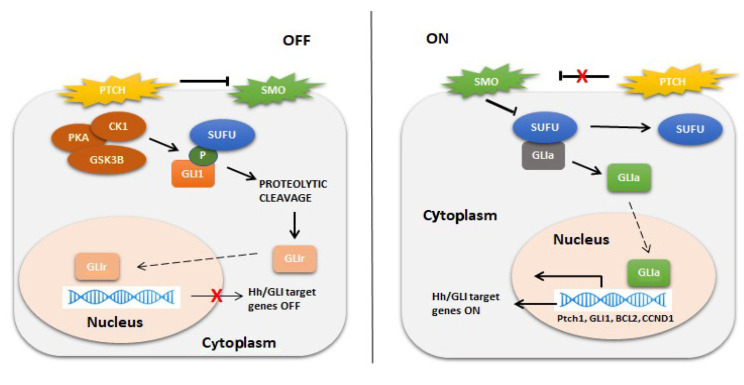
Activation of the Hh/GLI signaling pathway. Left panel: In the absence of ligand binding, PTCH exerts repressive effects on SMO. GLI transcription factors are sequestered by SUFU and phosphorylated by PKA, CK1, and GSK3β, marking them for proteolytic cleavage. The cleavage of the C-terminal domain creates GLIr, the repressor form of the transcription factor. GLIr then translocates into the nucleus and represses the transcription of Hh/GLI target genes. Right panel: Hh ligand binding to the extracellular domain of PTCH inhibits the receptor, relieving the repressive effects on SMO. SMO then inhibits the sequestration by SUFU and phosphorylation by PKA, CK1, and GSK3β, sparing GLI from proteolytic cleavage. The fulllength form of GLI is a transcriptional activator that translocates into the nucleus and promotes the transcription of Hh/GLI target genes such as PTCH1, GLI1, BCL2, Cyclin D1, etc. *PTCH*: *Protein patched homolog*; *SMO*: *Smoothened*; *GLI1*: *glioma-associated oncogene 1*; *SUFU*: *Suppressor Of Fused Homolog*; *PKA*: *protein kinase A*; *CK1*: *Casein kinase 1*; *GSK3β*: *Glycogen Synthase Kinase 3 Beta*; *GLIr*: *the repressor form of the transcription factor GLI*; *Hh/GLI target genes*: *Hedgehog/glioma-associated oncogene target genes*; *GLIa*: *the transcriptional activator of GLI*; *PTCH1*: *Protein patched homolog 1*; *BCL2*: *B-cell lymphoma 2*.

*GLI1* belongs to a family of genes (*GLI1*, *GLI2*, and *GLI3*) that encode transcription factors, mediating the Hedgehog (Hh) signaling pathway (Figure 5). This pathway regulates cell growth and differentiation under normal conditions, but under aberrant conditions, it leads to tumorigenesis in a wide range of tumors, including basal cell carcinoma, gliomas, and pancreatic, colorectal, prostate, lung, and breast carcinomas. The transcription factors GLI1, GLI2, and GLI3 contain five conserved tandem C2-H2 zinc finger domains (ZF1–ZF5) and a histidine/cysteine consensus sequence between the zinc fingers. ZF2-ZF5 directly interact with DNA, while ZF1 does not do so directly [73,74]. In this way, the zinc finger domains that bind to DNA and the consensus sequences in their target genes can initiate or inhibit the transcription of Hedgehog pathway target genes. GLI activation can occur through different mechanisms (canonical activation, noncanonical activation through ubiquitination, and deacetylation) [73,74].

Two types of genetic alterations of *GLI1* have been described: (1) *GLI1* fusions affecting the *ACTB*, *MALAT1*, and *PTCH1* genes in a subgroup of soft tissue tumors with a characteristic nested monomorphic epithelioid morphology; (2) another group of soft tissue tumors that were morphologically similar but lacked canonical *GLI1* gene fusions and instead had *GLI1* amplifications [75,76,77,78,79,80,81].

Similar to the detection of *MDM2* gene amplifications, the most widely used techniques for detecting *GLI1* gene amplifications are IHC (immunohistochemistry) and FISH (fluorescence in situ hybridization). For the study of *GLI1* gene amplifications, the FISH technique is performed using a combination of two FISH probes. One probe marks the centromere of chromosome 12, serving as a control for ploidy (a control probe to determine the number of chromosome 12 copies per cell), and the other probe marks the region of interest (*GLI1*). The following FISH signal patterns are considered positive: (1) a region of homogeneous staining; (2) double minutes; (3) ring chromosomes; and (4) multiple small amplicons of various sizes, with a ratio of at least 10:1 with respect to the centromeric 12 reference [75,76,77,78,79,80,81].

## 5. Does Isolated *MDM2*, *CDK4*, or *GLI1* Amplification Matter vs. Chromosomal Region 12q13-q15 Amplification?

Amplification of genes in the chromosomal region 12q13-15 has been observed in various soft tissue tumors, with liposarcoma (LPS) being the most common adult sarcoma, accounting for nearly 20% of cases worldwide [56,77,82,83,84,85,86,87,88,89,90,91,92,93,94,95,96,97,98,99,100,101,102,103,104,105,106,107,108]. Distinguishing well-differentiated liposarcoma/atypical lipomatous tumors from benign lipomatous neoplasms and dedifferentiated liposarcomas from high-grade sarcomas can be challenging [93]. Cytogenetic studies have identified ring chromosomes or supernumerary markers composed of amplicons from 12q13-15, including the *MDM2*, *CDK4*, *GLI1* genes, among others [56,77,82,83,84,85,86,87,88,89,90,91,92,93,94,95,96,97,98,99,100,101,102,103,104,105,106,107,108]. These cytogenetic findings are relevant for interpreting various FISH amplification patterns, especially in liposarcoma variants [93]. However, it remains unclear what clinical and therapeutic implications the level of *MDM2* gene amplification alone may have and whether it is accompanied by the amplification of other genes in the same chromosomal region 12q13-15, such as *CDK4*, *GLI1*, *DDIT3*, or *STAT6* [56,77,82,83,84,85,86,87,88,89,90,91,92,93,94,95,96,97,98,99,100,101,102,103,104,105,106,107,108] (Figure 1). A study of dedifferentiated liposarcoma found that high levels of *MDM2* amplification (>38 copies) and *CDK4* amplification (>30 copies) were correlated with reduced disease-free survival (DFS) and disease-specific survival (DSS) [92].

*CDK4* gene amplification in sarcomas has been observed in both bone and soft tissue tumors, while *MDM2* gene amplification predominates in soft tissue tumors, often without coamplification of other genes in the 12q13-q15 region [77,91,92,93,94,95,96,97,98,99,100,101,102,103,104,105,106,107,108].

In a group of soft tissue tumors with shared morphological characteristics, two genetic alterations in *GLI1* have been described: *GLI1* fusions (in a low frequency of cases studied) and high-level amplifications, often coamplified with neighboring genes in the 12q13.3-q15 region, such as *DDIT3/CDK4/MDM2/STAT6* genes [75,76,77,78,79,80,81,104,105,106,107,108].

## 6. Implications of Detecting Isolated *MDM2*-*CDK4*-*GLI1* Alterations or Chromosomal Region 12q13-q15 Amplification in the Anatomopathological Differential Diagnosis of Mesenchymal Neoplasms

The chromosomal region 12q13-15 is rich in oncogenes and contains several genes involved in the pathogenesis of various mesenchymal neoplasms. Notable genes in this region include *MDM2, CDK4, STAT6, DDIT3,* and *GLI1* [56,77,82,83,84,85,86,87,88,89,90,91,92,93,94,95,96,97,98,99,100,101,102,103,104,105,106,107,108]. Amplification of *MDM2* and *CDK4* genes can be detected in various mesenchymal and nonmesenchymal neoplasms [56,77,82,83,84,85,86,87,88,89,90,91,92,93,94,95,96,97,98,99,100,101,102,103,104,105,106,107,108]. The presence of amplification in one or both of these genes can strongly suggest diagnoses such as well-differentiated liposarcoma, dedifferentiated liposarcoma, intimal sarcoma, or low-grade central or parosteal osteosarcoma [92,93,94,95,96,97]. However, it is important to note that these genes can also be amplified in other tumor types, such as carcinomas or melanomas [98,99,100]. Therefore, gene amplification alone is not entirely specific for making a definitive diagnosis and requires the integration of clinical, radiological, morphological, and immunohistochemical findings.

Intrachromosomal rearrangements involving *STAT6* are characteristic molecular alterations observed in solitary fibrous tumors [101,109,110,111]. On the other hand, the *DDIT3* gene is known to be rearranged in myxoid/round cell liposarcomas, which can fuse with either *EWSR1* or *FUS*, and these fusions serve as diagnostic markers for this entity [112]. The amplification of *DDIT3* and *STAT6* may vary depending on the amplicon length in cases of mesenchymal neoplasms with 12q13-15 amplification [101,102,103,104,105,106,107,108,109,110,111,112,113,114,115].

Recently, a group of mesenchymal neoplasms with predominantly epithelioid morphology has been described [75,76,77,78,79,80,81,104,105,106,107,108]. These neoplasms typically express CD56, S100, and p16 and are predominantly located in the head and neck region, displaying alterations in the *GLI1* gene. *GLI1* is situated in the 12q13.3 region, in close proximity to the genes within the 12q13-15 region, particularly *DDIT3*. Neoplasms with *GLI1* alterations may exhibit either GLI1 rearrangements or amplifications of this gene [75,76,77,78,79,80,81,104,105,106,107,108].

In cases with *GLI1* amplification, it is not unusual to observe coamplification of *DDIT3*, *CDK4*, *MDM2,* and/or *STAT6* [75,76,77,78,79,80,81,104,105,106,107,108]. This coamplification can lead to nuclear and/or cytoplasmic immunohistochemical expression of DDIT3, CDK4, MDM2, and/or STAT6 in these *GLI1*-altered tumors, with the extent of expression depending on the size of the amplicon [104,105,106,107,108].

Similarly, well-differentiated/dedifferentiated liposarcomas and other neoplasms with *MDM2*/*CDK4* amplification may also exhibit coamplification of *GLI1*. This coamplification can result in nuclear and/or cytoplasmic expression of GLI1, as detected by immunohistochemistry [104,105,106,107,108]. This genetic overlap can pose diagnostic challenges, particularly when dealing with small cylindrical biopsies where clinicopathological correlation may be limited.

Consider a scenario where a small cylindrical biopsy, providing limited material, is obtained from a neoplasm situated in an atypical location for liposarcoma. This neoplasm exhibits an epithelioid morphology and phenotypic expression of MDM2/CDK4/p16, alongside *MDM2/CDK4* amplification, as confirmed by FISH. In such cases, dedifferentiated liposarcoma might be a potential diagnosis due to the presence of *MDM2/CDK4* amplification. However, because the neoplasm is located in an atypical location for liposarcoma, the possibility of a neoplasm with *GLI1* alteration, particularly *GLI1* amplification, should also be considered. Neoplasms with *GLI1* alterations often feature an epithelioid morphology and can coamplify other genes like *MDM2/CDK4* [104,105,106,107,108].

It is important to note that many liposarcomas and other sarcomas, including intimal sarcoma with *MDM2/CDK4* amplification, may exhibit cytoplasmic and/or nuclear expression of STAT6 without necessarily indicating a solitary fibrous tumor [92,93,94,95,101]. These tumors can also demonstrate cytoplasmic and/or nuclear immunohistochemical expression for GLI1 without confirming the presence of a primary neoplasm with *GLI1* alteration. Therefore, it is critical to correlate all clinical, radiological, morphological, phenotypic, and molecular findings to arrive at a definitive diagnosis. This means that when dealing with a large retroperitoneal tumor that expresses and/or amplifies *MDM2*, the initial consideration should be liposarcoma rather than a neoplasm with *GLI1* alteration. Conversely, if a tumor is located in the head and neck or in an atypical liposarcoma location and shows *MDM2* amplification, the possibility of a neoplasm with *GLI1* alteration should be systematically ruled out.

Another crucial aspect in the diagnosis of neoplasms with *GLI1* alterations is that while amplification is reliably detected by FISH, rearrangement is typically identified through genomic sequencing (NGS, RNAseq), which is not universally accessible. Given the proximity of the *DDIT3* gene to *GLI1*, the use of a *DDIT3* split probe may be useful in identifying the possibility of *GLI1* rearrangement in neoplasms that display a phenotype or morphology typical of a neoplasm with *GLI1* alteration [104,105,106,107,108].

**Figure 6:**
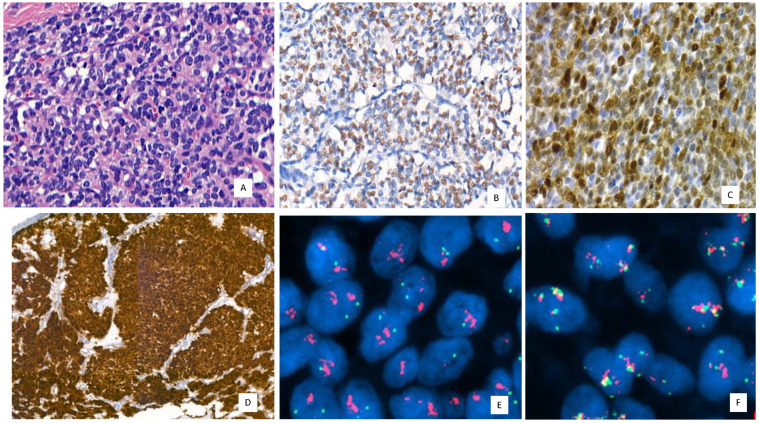
*GLI-1*-amplified neoplasm with *CDK4* coamplification. (**A**) Round and epithelioid cell neoplasm with isolated mitoses and low-grade morphology. Hematoxylin and eosin (H and E) 400×, (**B**) GLI1 immunoreactivity with moderade and nuclear expression 200×. (**C**) CDK4 moderates and diffuses nuclear immunoreactivity 400×. (**D**) Strong and diffuse p16 immunoexpression in GLI1 with nuclear and cytoplasmic stain, 200×, (**E**,**F**) *GLI1* (red signals/*GLI1* and green signals centromeric region) and *CDK4* (red signals/*CDK4*, green signals centromeric region, yellow signals/signal overlaps/green and red) coamplification by FISH. Magnification 63×.

The correlation between immunohistochemical expression for MDM2 and the presence of *MDM2* amplification by FISH is not always perfect. In fact, there are cases where MDM2 is only focally positive or even negative by immunohistochemistry, despite the confirmation of *MDM2* amplification by FISH. This emphasizes that FISH remains the ideal technique for detecting this genetic alteration. In a recent series of adipocytic tumor cases reported in a study by Vargas et al. [113], later confirmed by Machado et al. [103], instances of well-differentiated liposarcomas with isolated nuclear atypia were identified. Despite this, they exhibited immunohistochemical expression for MDM2/CDK4 and p16, alongside confirmation of *MDM2* amplification by FISH [103,113]. Similar situations can arise in neoplasms with *GLI1* alterations (Figure 6), where immunohistochemistry for GLI1 is not always sufficient, necessitating molecular confirmation [104,105,106,107,108].

Despite the diagnostic implications that the overlap of genetic alterations in neoplasms with changes in genes within the 12q13-15 region could create, the discovery of coamplifications of *MDM2* with *CDK4* and *GLI1* offers new therapeutic targets in neoplasms with MDM2/CDK4 amplification [92,93,94,95,96,97,98,99,104,105,106,107,108]. This particularly applies to well-differentiated or dedifferentiated liposarcomas, which might not only benefit from MDM2 or CDK4 inhibitors, but also from anti-GLI1 therapies. Similarly, neoplasms with *GLI1* amplification and coamplification of *MDM2/CDK4* could benefit from MDM2/CDK4 inhibitors.

Lastly, it is worth noting that *MDM2* or *CDK4* amplification is not exclusive to mesenchymal neoplasms; this genetic alteration has also been observed in other epithelial neoplasms or melanomas [98,99,100]. This suggests the potential use of MDM2 or CDK4 inhibitors in neoplasms where alterations in these genes do not aid the pathological diagnosis but may help identify potential therapeutic targets.

### 6.1. Therapeutic Implications

#### 6.1.1. MDM2 Inhibitors

The *MDM2* gene inhibits the activity of the p53 tumor suppressor protein. Amplification of *MDM2* hinders p53 function, thereby compromising its protective role [114,115,116,117,118].

*MDM2* amplification is known to occur in various types of tumors, including sarcomas, carcinomas, and melanomas. Consequently, drugs designed to inhibit the MDM2-p53 interaction have the potential to restore wild-type p53 function, leading to cell cycle arrest and apoptosis induction [115,116,117,118].

Currently, anti-MDM2 drugs are in the developmental phase, undergoing clinical trials with promising results, especially for tumors that are inoperable, advanced, metastatic, aggressive, and associated with a poor prognosis, such as dedifferentiated liposarcoma (DDLPS), for instance. The Brightline-1 clinical trial is assessing the efficacy and tolerability of BI 907828, an MDM2 antagonist, which has shown encouraging results in DDLPS during Phase I development [119,120].

The rationale for developing BI 907828 is based on preclinical models that demonstrated a response in mice with DDLPS xenografts bearing *MDM2* amplification. This drug exhibits a favorable safety profile, with reported toxicities in 88% of patients. The most common side effect was nausea (66%), and 41% of patients experienced equal to or greater than grade 3 toxicity, primarily including neutropenia (20%), thrombocytopenia (19%), and anemia (10%) (113). BI 907828 is an orally administered drug with a long half-life and is being evaluated in various types of sarcomas characterized by MDM2 overexpression [119,120].

The evidence accumulated during Phase I of the Brightline-1 clinical trial supports the further development of the drug, justifying the ongoing Phase II/III studies. The primary objective of the study is to compare two different doses of BI 907828 (30 mg/21 days vs. 40 mg/21 days) with doxorubicin 75 mg/m2/21 days. The primary endpoint is progression-free survival (PFS). The results of this clinical trial will determine whether the standard clinical practice for treating DDLPS and potentially other sarcomas characterized by MDM2 overexpression can be improved [118,119,120,121]. MDM2-inhibitors ongoing clinical trials are summarized in Table 1.

#### 6.1.2. CDK4/6 Inhibitors

The cyclin pathway can be disrupted in 1 out of every 4 sarcomas and is pathognomonic for some very specific types of sarcomas. This suggests that targeting this pathway could offer a promising treatment option for certain patients with advanced or metastatic sarcomas that have progressed beyond standard therapies. These patients often face limited treatment possibilities, resulting in a poor prognosis and reduced survival [121,122].

The availability of CDK4 and CDK6 cyclin inhibitor drugs (anti-CDK4 and anti-CDK6) makes them excellent choices for targeting sarcomas with these genetic alterations in the form of deletions or amplifications [121,122].

Currently, there are three cyclin inhibitors available: Palbociclib (Ibrance^®^, Pfizer), Ribociclib (Kisqali^®^ Novartis), and Abemaciclib (Verzenios^®^ Eli Lilly) [123,124,125,126]. All three of these drugs have been approved by the EMA for the treatment of metastatic breast cancer. It is important to note that only Abemaciclib can be used for adjuvant treatments in breast cancer, typically in combination with hormone therapy.

While we lack extensive information on the efficacy of cyclin inhibitors in sarcoma treatment, the available data suggest that this class of drugs could offer a promising new treatment option for patients with advanced sarcomas [83,127,128,129,130,131,132,133,134,135].

Dedifferentiated liposarcomas (DDLPS) represent a particularly aggressive type of sarcoma known for its high recurrence rate after surgical resection, rapid growth, and poor prognosis. These tumors often exhibit overexpression of CDK4, a characteristic also found in myxoid liposarcomas (MLPS), which is another form of liposarcoma associated with a high likelihood of recurrence and rapid growth [83,127]. Ongoing clinical trials are investigating the use of Palbociclib with varying dosing intensities, and the results show no significant differences in progression-free survival (PFS) at 12 weeks (57% vs. 66%) or in median PFS (17.9 weeks vs. 18 weeks [128,129].

It is important to note that, as of today, none of these cyclin inhibitors have received approval for sarcoma treatment. Research is actively continuing with several cyclin inhibitors in the clinical development phase.

In both Phase I and Phase II clinical trials, ribociclib has demonstrated efficacy in terms of inducing a response or prolonged stabilization [131,132,133]. However, combining the cyclin inhibitor with an MDM2 inhibitor did not result in improved outcomes for patients with DDLPS and WDLPS [133,134].

Based on these preliminary results, it is reasonable to conclude that cyclin inhibitor drugs may play an important role in treating DDLPS with *CDK4/6* amplification. Nevertheless, further investigation is needed to determine whether quantifying CDKN2A/p16 levels can serve as a useful prognostic biomarker for WDPLS and DDLPS [133,134]. This is of particular interest because p16 overexpression is a common feature in well-differentiated and dedifferentiated liposarcomas and is often relied upon by pathologists for diagnostic purposes.

Leiomyosarcomas (LMS) are one of the most common histological types of sarcomas, and alterations in the cyclin pathway are present in 1 out of every 5 patients with this tumor [135]. Cyclin pathway inhibitor drugs have demonstrated the ability to inhibit the growth of uterine LMS cell cultures. In fact, it has been observed that the genetic profile of uterine LMS with mutations in *BCOR* shows amplifications of *MDM2* and *CDK4*, among others, which is reminiscent of the genetic profile of DDLPS. By extrapolation, it could be hypothesized that CDK4/6 inhibitors might be therapeutically effective in these uterine LMS cases with *BCOR* fusion, similar to their effectiveness in DDLPS [135,136,137,138].

When assessing the effectiveness of cyclin inhibitor drugs in nonuterine LMS, contradictory results have been reported [138], leading to the development of clinical trials aimed at clarifying this issue [128]. The role of cyclin inhibitors in advanced gastrointestinal stromal tumors (GIST) with CD117 mutations is not yet clearly defined [139].

Genetic alterations observed in osteosarcomas (OS) [139] also suggest that drugs capable of blocking cyclin activity could play a role in the therapeutic strategy for these bone sarcomas. However, so far, the lack of clinical trials makes it challenging to demonstrate their efficacy.

Regarding another rare tumor, alveolar rhabdomyosarcomas (RMS), amplifications at 12q13q14 and 2p24 have been described, leading to the overexpression of CDK4 and CDK6, among other genes [140]. Clinical trials are currently underway to help define the role of CDK4 inhibitors in RMS. In contrast, synovial sarcomas (SS) frequently exhibit overexpression of CDK2, CDK4, and MDM2, loss of *CDKN2A,* and mutations in genes such as *CDK 4/6* [141]. Similarly, intimal sarcomas exhibit a much higher frequency of *MDM2/CDK4* amplification, which serves as a valuable diagnostic tool for pathologists [92,93,94,95].

In other sarcomas with entirely different histologies, such as epithelioid hemangioendothelioma [142] or high-grade chondrosarcomas [143], genetic alterations affecting CDK4 and CDK6 have also been described. This could potentially position CDK4/6 inhibitors as targeted drugs for these indications. In such cases, this potential indication could be extended to any type of sarcoma, with alterations in the regulation of CDK4 and CDK6.

Table 2 summarizes the published clinical trials with CDK4 inhibitors.

#### 6.1.3. GLI1 Inhibitors

Aberrant activation of *GLI1* in tumors is associated with cell proliferation and survival, angiogenesis, metastasis, metabolic pathway alterations, and chemotherapy resistance [146,147]. Understanding the mechanisms of GLI action may lead to the development of biomarkers that can inhibit GLI activity, providing clear therapeutic benefits to patients with different types of cancer that exhibit alterations in the pathways in which these proteins are involved.

The activation of *GLI* genes can occur through canonical or noncanonical mechanisms, with the RAS-RAF-MEK-ERK pathway (Figure 5) being the most frequently involved in noncanonical mechanisms and responsible for the proliferation of colon cancers, as is the PI3K-AKT-mTOR pathway in pancreatic cancer [147,148,149].

In the case of sarcomas, it has been shown that GLI1 and GLI2 can be overexpressed in various types of sarcomas, both in bone (osteosarcomas) and soft tissue (rhabdomyosarcomas) [148,149,150].

Given all of the above, the possibility of inhibiting GLI1 and GLI2 may have beneficial effects in the treatment of all tumors with activation of these factors by suppressing the Hh activation pathway [147,150].

Currently, there is limited information that allows us to understand the actual effect of GLI1 and GLI2 inhibition, although it has been demonstrated that GLI1/2 inhibitors can block tumor proliferation in mice with human tumor xenografts [150].

The action mechanism of these inhibitory proteins is to reduce effector proteins of the Hh and AKT/mTOR signaling pathways. Their efficacy seems to improve when combined with mTOR pathway inhibitors (Temsirolimus or Rapamycin) or mitosis inhibitors such as vincristine.

The availability of GLI inhibitors is currently very limited, although several, such as JC19, are showing promising therapeutic activity in preclinical development phases. However, they face the challenges of metabolic instability, low solubility, and high hydrophobicity, which pose significant obstacles to their viable clinical development [149].

Other GLI inhibitors/antagonists currently in development include GANT 61 (GLI1/2 inhibitor), GANT 58 (GLI1 transcription inhibitor antagonist), Globascione B (GLI1 inhibitor), GLI antagonists-1 (GLI1 antagonist), and TPB15 (an oral form that blocks GLI1) [151].

In conclusion, although the amplification of *MDM2* and *CDK4* has therapeutic implications [128,129,130,131,132,133,134,144,145,152], these alterations are entirely nonspecific to any particular diagnosis, and a thorough clinical, histological, and phenotypic correlation should be conducted. *GLI1*-altered neoplasms may exhibit morphological overlap with certain mesenchymal neoplasms and may also have coamplification of neighboring genes, depending on the amplicon length. While this may pose challenges in the differential diagnosis, it also opens the window to new therapeutic approaches in these novel tumors.

## Figures and Tables

**Table 1:** MDM2-inhibitors.

Target	Drug	Phase	Sarcoma	Combination	NCT	Published
MDM2	Navtemadlin (AMG-232 [KRT-232	1B	Only	Radiotherapy	NCT03217266	
MDM2	Navtemadlin	1/2	NO	TL-895	NCT02825836	
MDM2	Navtemadlin	2	No	No	NCT03662126	
MDM2	APG-115	2	No	nO	NCT03781986	
MDM2	Brigimadlin (BI 907828	2	DDLPS	No	NCT06058793	
MDM2	Brigimadlin BI 907828	2	DDLPS	ADRYAMICIN	NCT05218499	
MDM2	Brigimadlin BI 907828	1	included	Ezabenlimab	NCT03964233	
MDM2	Brigimadlin BI 907828	1	Included	No	NCT03449381	LoRusso [119,120]
MDM2	CGM097	1B	included	NO	NCT01760525	
MDM2/MDMX	Idasanutlin	1B	RMS	Selinexor	NCT05952687	
MDM2/MDMX	Idasanutlin	1/2	No	Atezolizumaband Cobimetinib	NCT03566485	

DDLPS: dedifferentiated liposarcoma, RMS: rhabdomyosarcoma.

**Table 2:** CDK4-inhibitors.

Reference	Phase	Drug	Sarcoma	Population	Effcicacy (R Rate o PFS)
Razak [133]	1b	Siremadlin and Ribociclib	Liposarcoma	74	3/74 (4%)
Dickson [128]	2	**Palbociclib**	Lipsarcoma	30	1/29 (3%)
Dickson [129]	2	Palobociclib	Liposarcoma	60	1/60 (2%) PFS 4 mo 57%
Shulman [144]	2	**Palbociclib and Ganitumab**	Ewing Sarcoma	10	0% PFS 6 mo 30%
Martin Broto [145]	2	Palbociclib	Advanced sarcoma not LPS (CD4A CDKN2A favorable)	23	0% PFS 6 mo 29%
